# Evaluating PurpleAir Sensors: Do They Accurately Reflect Ambient Air Temperature?

**DOI:** 10.3390/s25103044

**Published:** 2025-05-12

**Authors:** Justin Tse, Lu Liang

**Affiliations:** Department of Landscape Architecture and Environmental Planning, University of California, Berkeley, CA 94720, USA; j_tse@berkeley.edu

**Keywords:** low-cost sensor calibration, crowdsourced data, environmental monitoring, heat wave

## Abstract

**Highlights:**

**What are the main findings?**

- PurpleAir sensors exhibit strong temperature overestimations with an *MAE* of 4.71 °C and *RMSE* of 6.30 °C.
- Sensor performance demonstrates nonlinear behavior with significant seasonal and diurnal variations.

**What is the implication of the main finding?**

- Calibrated PurpleAir sensors have the potential to advance hyperlocal heat mapping and multi-hazard vulnerability assessments.

**Abstract:**

Low-cost sensors (LCSs) emerge as a popular tool for urban micro-climate studies by offering dense observational coverage. This study evaluates the performance of PurpleAir (PA) sensors for ambient temperature monitoring—a key but underexplored aspect of their use. While widely used for particulate matter, PA sensors’ temperature data remain underutilized and lack thorough validation. For the first time, this research evaluates their accuracy by comparing PA temperature measurements with collocated high-precision temperature data loggers across a dense urban network in a humid subtropical U.S. county. Results show a moderate correlation with reference data (*r* = 0.86) but an average overestimation of 3.77 °C, indicating PA sensors are better suited for identifying temperature trends but not for precise applications like extreme heat events. We also developed and compared eight calibration methods to create a replicable model using readily available crowdsourced data. The best-performing model reduced *RMSE* and *MAE* by 51% and 47%, respectively, and achieved an *R*^2^ of 0.89 compared to the uncalibrated scenario. Finally, the practical application of PA temperature data for identifying heat wave events was investigated, including an assessment of associated uncertainties. In sum, this work provides a crucial evaluation of PA’s temperature monitoring capabilities, offering a pathway for improved heat mapping, multi-hazard vulnerability assessments, and public health interventions in the development of climate-resilient cities.

## 1. Introduction

The growing demand for high spatial-temporal urban environmental data, driven by climate-resilient city initiatives, has rapidly advanced the development of low-cost sensors (LCSs). Traditionally, localized environmental data were acquired from regulatory stations with spatial-temporal limitations in capturing complex urban phenomena [1]. LCS offers a promising alternative by filling gaps in regulatory monitoring networks, bringing transformational benefits [2,3], such as motivating scientific communities to better understand hyperlocal problems [4,5] and empowering public environmental awareness [6].

Among the various applications of LCS, air quality monitoring constitutes a significant portion of large-scale projects such as AirCasting, Citi-Sense, AirVisual, and City Health Outlook [7,8]. PurpleAir (PA), a leading air quality LCS vendor, stands out with over 30,000 citizen scientists contributing to its real-time map (as of July 2024). With over 18 times more devices than government-operated weather stations in the U.S. [9], PA enables unprecedented block-level environment characterization [10,11,12]. Unlike remote sensing technologies that provide proxy data [13,14], PA provides high temporal resolution ground-level in situ measurements reflecting direct human environmental exposure.

While most studies focus on PA sensors for particulate matter measurements, these devices simultaneously collect valuable meteorological data—including temperature, relative humidity (RH), and pressure—which have primarily been used for detecting sensor malfunctions and supporting field calibration [15]. As demand grows for integrated heat health information systems, large-scale sensor networks for measuring ambient air temperature have become critical for improving urban heat island (UHI) monitoring and modeling to support the development of climate-resilient communities [16]. Ongoing efforts to enhance UHI mapping and develop a comprehensive national heat–health information system position PA sensors as a potentially pivotal platform for raising public awareness and strategizing interventions. Furthermore, the concurrent collection of air quality and temperature data enables researchers to explore the multi-disaster vulnerability in urban systems, such as the intricate relationship between UHI and urban pollution islands and their mechanisms [17,18]. As the majority of these studies relied on remote sensing products or in situ observations from sparely distributed stations [19], PA sensors present the opportunity to overcome this limitation and reveal hyperlocal patterns for these urban phenomena.

Despite their significant implications in urban climatology and public health, data quality remains an indispensable concern in LCS applications [20,21,22]. Many studies use field calibration techniques by collocating LCS with regulatory instruments to adjust biases and improve accuracy [23,24,25]. While there is a growing interest in PA’s air quality monitoring performance, thorough assessments of PA’s temperature data remain limited, with only a few intercomparison studies [26], which differ from field evaluations. Couzo et al. evaluated PA temperature measurements using a collocated research-grade instrument in Asheville, North Carolina, and developed a simple linear regression correction model [27]. However, their study did not evaluate the spatial and temporal variability in sensor performance. This information is critical for holistically understanding influential factors of measurement biases to improve field calibration methods. Furthermore, PA recommends a constant correction of −4.4 °C, hereinafter PA-suggested calibration, to align with ambient conditions [28]. To the best of our knowledge, this black box factory adjustment has not been empirically tested in the ambient environment. The performance of electrical devices can be sensitive to various mechanical and ambient factors [29], necessitating a comprehensive understanding of PA’s performances and the underlying influencing factors. As emerging UHI studies increasingly rely on PA temperature data to supplement satellite or regulatory station observations [30], addressing these uncertainties has become critical.

This study offers a first-hand evaluation of PA’s temperature monitoring capabilities and develops a calibration approach through three key objectives: (1) to assess PA’s performance in estimating ambient temperature using collocated high-accuracy temperature data loggers across a dense sensor network; (2) to evaluate PA temperature data uncertainties through the application of heat wave detection; and (3) to develop and compare calibration methods to create a replicable model based on widespread crowdsourced data.

## 2. Materials and Methods

### 2.1. Collocated Temperature Sensor Network and Data Preprocessing

The PA sensors and reference data were collected from an LCS network of 51 sites in Denton County, Texas, where each site was equipped with collocated PA-II-SD sensors and Onset HOBO MX2301A temperature/relative humidity (*RH*) outdoor data loggers. PA-II-SD sensors use Plantower PMS5003 laser-based particulate sensors to measure PM_1_, PM_2.5_, and PM_10_ concentrations, along with a BOSCH BME280 sensor for pressure, temperature, and humidity. Data are transmitted via a 2.4 GHz 802.11 b/g/n wireless network, with an SD card available for local storage. The Onset HOBO MX2301A is a rugged, battery-powered temperature and RH data logger designed for outdoor environmental monitoring. The temperature sensor has a reported accuracy of ±0.2 °C and ±2% and a drift of less than 0.01 °C and 1% per year [31], making it a reliable reference for this study. MX2301A uses Bluetooth low energy for data retrieval via mobile devices. We configured the HOBO data loggers to record temperature at 30 min intervals, balancing data resolution needs and battery life, from March 2022 to August 2023. This period includes the record-hot summer of 2023, offering key insights into PA sensor performance during prolonged extreme heat. All collocated sensors were installed at 1.5 to 2 m above ground level to ensure comparable measurables that are representative of the local scale [32]. PA sensor mounting locations were carefully selected to prevent overheating of optical components, ensure adequate airflow, and maintain stable access to power and WiFi. In residential areas, sensors were typically mounted on structures like yard posts that are fully shaded yet positioned slightly away from walls to minimize interference from wall material reflectance. HOBO data loggers, housed in solar radiation shields, are more flexible in placement as they can be exposed to direct or reflect sunlight. They were collocated as closely as possible to the PA sensors, with the only constraint being a minimum distance of 2 m from nearby tree canopies to reduce the influence of evapotranspiration (Figure S1).

**Figure 1:**
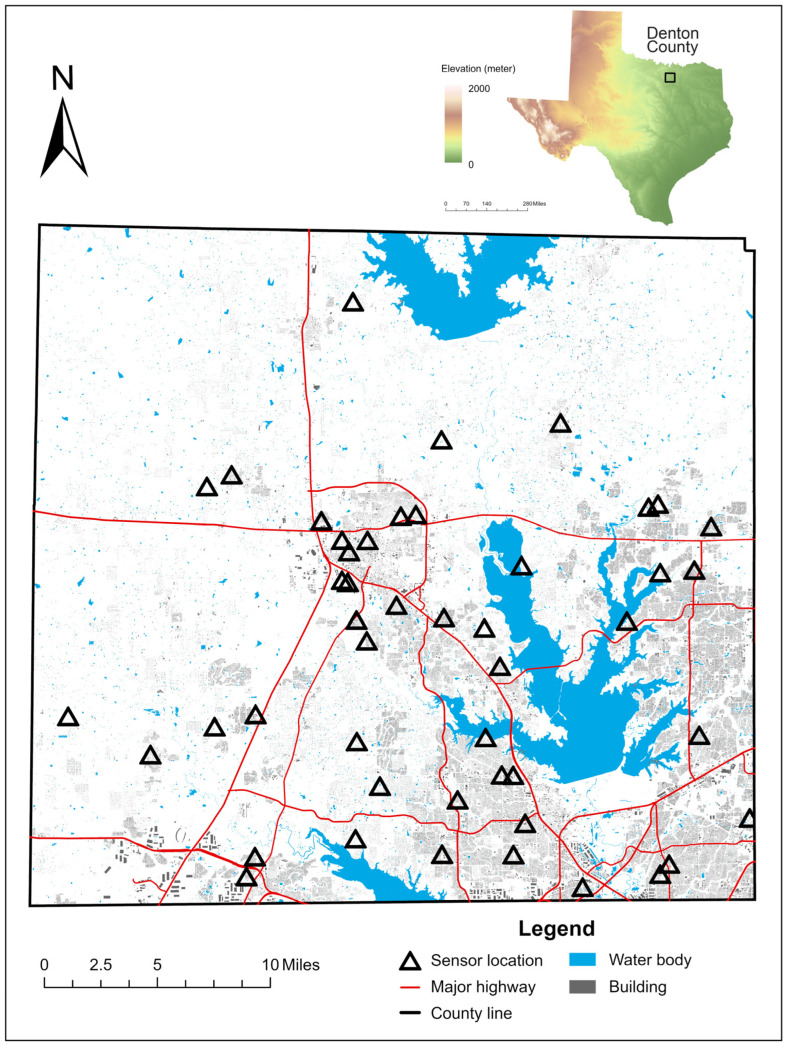
The distribution of sensors. Each site has a pair of a collocated PurpleAir sensor and HOBO data logger.

Denton County’s landscape comprises predominantly grass (50.6%), tree canopy (20.4%), and water bodies (8.2%), with an elevation ranging from approximately 130 to 300 m. The region’s humid subtropical climate is characterized by humid, hot summers and cool winters, with year-round precipitation. To ensure optimal spatial representation, we employed a stratified sampling approach and used 300 × 300 m grid cells as the basic spatial unit, dividing the area into six urban strata based on proximity to major traffic and the percentage of impervious surfaces (Table S1) [5]. The 51 sites were distributed across 4 Rural High, 12 Rural Low, 1 Suburban High, 16 Suburban Low, 6 Urban High, and 12 Urban Low sites, ensuring comprehensive spatial coverage (Figure 1).

### 2.2. Sensor Data Preprocessing

The raw data from PA and HOBO sensors were first averaged to hourly measurements, represented by *T_PA_* and *T_HOBO_*, to capture the diurnal temperature cycles. Datasets were then matched by sensor locations and timestamps for pairwise comparisons, yielding 282,355 pairwise measurements. After that, z-scores were calculated for every PA and HOBO temperature observation in the joint dataset, and a z-score threshold of two was applied to all measurements across all time to exclude outliers in HOBO and PA measurements. This ensured valid measurements from both instrument types during the study period to avoid biased comparison due to sensor malfunctions or measurement errors from either collocated sensor. Finally, two sites with limited sample sizes were removed to ensure statistical significance, yielding a total number of pairwise observations of 261,310 measured from 47 sites.

### 2.3. Spatial-Temporal Variations in Sensor Performance

We performed spatial-temporal analysis on sensor performance to better understand its behavior across diverse urban environments and seasonal conditions. Spatially, measurements were grouped based on the six strata to identify patterns of systematic differences, accounting for varying impacts from anthropogenic heat output, urban heat intensity, and modification of airflow. We calculated the inter- and intra-group differences using temperature anomalies (*dT*), which are expressed as:

(1) $${dT}_{i}=T_{PA,i}{-T}_{HOBO,i}$$

where $T_{PA,i}$ and $T_{HOBO,i}$ are pairwise measurements from PA and HOBO sensors for observation *i*, with *i* ranging from 1 to *n* = 261,310.

We tested the significance of inter- and intra-strata differences using the Kruskal–Wallis H-test, chosen for its suitability with non-normal distributions and variance dissimilarities identified via the Kolmogorov–Smirnov test and Levene’s test [33]. The null hypothesis is that there is no in-class variability across the strata due to similar thermal climates. The Suburban High group was omitted from the intra-group test due to insufficient sensor samples. The inter-group evaluation was followed up by a nonparametric post hoc pairwise test (Dunn’s test) to identify specific disparities.

Temporally, all sensor data were grouped by month of the year and hour of the day to examine seasonal and diurnal variations, accounting for potential nonlinear behavior at extreme temperatures caused by ambient heat and sensor self-heating during operation [34].

### 2.4. Performance Metrics

We selected the Pearson correlation coefficient (*r*), coefficient of determination (*R*^2^), mean bias error (*MBE*), mean absolute error (*MAE*), and root mean square error (*RMSE*) as performance metrics. *MBE* measures the average differences between predicted and observed values, suggesting overestimation or underestimation. *MAE* indicates the total error values, whereas *RMSE* is more sensitive to larger deviations.

(2) $$MBE=\frac{1}{n}\sum_{i=1}^{n}{dT}_{i}$$

(3) $$MAE=\frac{1}{n}\sum_{i=1}^{n}\left|{dT}_{i}\right|$$

(4) $$RMSE=\sqrt{\frac{1}{n}\sum_{i=1}^{n}{\left({dT}_{i}\right)}^{2}}$$

### 2.5. Independent Variables

We developed field calibration models for PA temperature sensors based on the protocol used for PM sensors [26,35]. Field calibration involves comparing collocated data collected from ambient environment and using data-driven approaches, such as statistical or machine learning models, to minimize discrepancies in sensor readings. While some studies employ complex machine learning models [36,37], we chose linear regression and multiple linear regression (MLR) for their model transparency, interpretability, and replicability, and they have proven reliable in studies requiring minimal parameter tuning and model selection [29].

We selected a set of meteorological factors as independent variables to account for the influence of ambient environmental conditions (Table 1). PA relative humidity (*RH_PA_*) has been proven as a useful factor in particulate matter measurements calibration to adjust for the hygroscopicity of particles [38,39]. Wind speed (*WNDS*) can induce biases in PA’s particle readings either high or low by a factor of 1.6 [40], but its influence on heat exchange between the environment and LCS has not been thoroughly explored yet. Hourly wind speed observations were acquired at the Denton Airport South ambient air monitoring station administered by the Texas Commission on Environmental Quality. Furthermore, radiative fluxes estimate incoming and outgoing solar radiation, serving as proxies for the time of day and sky conditions. Hence, both downwelling longwave (*LW*) and shortwave (*SW*) irradiance were included to account for the effects of cloudy days on temperature variability [41]. Hourly *LW* and *SW* were acquired from the GOES-EAST Surface Solar Irradiance product provided by the EUMETSAT Ocean and Sea Ice Satellite Application Facility [42]. We extracted *LW* and *SW* values at each site based on its coordinates.

### 2.6. Developing Calibration Models

We first conducted overall and monthly bivariate analyses between hourly *dT* and each meteorological factor in Table 1 for variable selection. The correlation coefficient (*r*) was used to assess the directions and strength of the relationship and the *p* value determined their significance. Only variables with a significance level of *p* < 0.001 were included as inputs for the calibration model development.

Eight models were developed using different combinations of additive terms and interactive terms to account for interdependence between variables [35]. These models were evaluated using the performance metrics described in Section 2.3 and the “leave one out” approach by splitting 20% of the data as a test set. The best calibration model was determined by considering the *R*^2^, *RMSE*, *MAE*, and Akaike information criterion (AIC) score to balance the model performance and complexity. An AIC score measures the goodness of fit while penalizing model complexity to reduce the risk of overfitting and underfitting, making it an ideal metric for model selection.

Model 1: Simple linear regression
  (5) $$T_{HOBO}={s_{1}T}_{PA}+\varepsilon$$
Model 2: MLR with an additive *RH_PA_* term
  (6) $$T_{HOBO}={s_{1}T}_{PA}+{s_{2}RH}_{PA}+\varepsilon$$
Model 3: MLR with an additive *SW* term
  (7) $$T_{HOBO}={s_{1}T}_{PA}+s_{2}SW+\varepsilon$$
Model 4: MLR with an additive *LW* term
  (8) $$T_{HOBO}={s_{1}T}_{PA}+s_{2}LW+\varepsilon$$
Model 5: MLR with additive *SW* and *LW* terms
  (9) $$T_{HOBO}={s_{1}T}_{PA}+{s_{2}LW+s}_{3}SW+\varepsilon$$
Model 6: MLR with additive *RH_PA_*, *LW*, and *SW* terms
  (10) $$T_{HOBO}={s_{1}T}_{PA}+{s_{2}RH+s}_{3}LW+s_{4}SW+\varepsilon$$
Model 7: MLR with additive *RH_PA_*, *LW*, *SW*, and *WNDS* terms
  (11) $$T_{HOBO}={s_{1}T}_{PA}+{s_{2}RH+s}_{3}LW+s_{4}SW+s_{5}WNDS+\varepsilon$$
Model 8: MLR with additive and multiplicative *T_PA_* and *RH_PA_* terms
  (12) $$T_{HOBO}={s_{1}T}_{PA}+{s_{2}RH}_{PA}+{s_{3}T_{PA}RH}_{PA}+\varepsilon$$

Since *dT* is likely to exhibit varying temporal patterns due to the oscillating influence of meteorological factors, we assess how much of its variance can be explained by the meteorological factors, after accounting for their deviations from observed monthly and diurnal patterns. We define the monthly diurnal deviations of *dT* as the anomaly *dT* (*dT*′), calculated as:

(13) $$\bar{{dT}_{mh}}=\frac{1}{N_{mh}}\sum_{k=1}^{N_{mh}}{dT}_{mn(K)}$$

(14) $$dT^{\prime }=dT-\bar{{dT}_{mh}}$$

where *N_mh_* is the number of observations in month *m* at hour *h* and ${dT}_{mn(K)}$ is the value of *dT* at the *k*th observations in month *m* at hour *h*.

Similarly, we define *T_HOBO_′*, *RH_HOBO_′*, *SW′*, *LW′*, and *WNDS′* as the anomalies of their respective variables, which are used as the independent variables in the MLR to model *dT′*. This anomaly model helps us determine the effectiveness of anomaly variables in calibrating *T_PA_*. We then created Model 9 by incorporating the anomaly corresponding to the variables in the best-performing model from Model 1 to Model 8. This adjustment accounts for unexplained errors when relying solely on the hourly observed values of the meteorological variables in Table 1. Finally, we compared the performance of Model 9 and the best model from Model 1 to Model 8 to determine the final calibration model. A variance inflation factor (VIF) was calculated for each independent variable in the final model to detect the severity of multicollinearity.

### 2.7. Performance in Apparent Temperature Calculation

Other than air temperature, we evaluated PA’s performance in measuring apparent temperature, commonly represented by the Heat Index (HI). HI is a better indicator of human heat exposure, as it reflects the temperature perceived by humans by combining air temperature, RH, and sometimes wind speed [43]. Given the significant implications of HI in public health and urban heat research [44,45], we anticipate that HI will be a key metric in studies involving PA sensors in public health or related fields. Here, we present the first assessment of PA sensors’ performance in estimating HI. We used an extended Heat Index that extends the calculation of the Heat Index to a wider range of temperature and RH [46], accommodating the high heat and humidity over Texas in the summer of 2023. HOBO- and PA-derived HIs were calculated using their corresponding meteorological data at the hourly interval. Subsequently, we compared their proportions in each HI class as defined by the National Weather Service to exemplify potential flaws in a potential application of PA temperature measurements.

## 3. Results and Discussion

### 3.1. Evaluation of Uncalibrated PA Temperature Measurements

**Figure 2:**
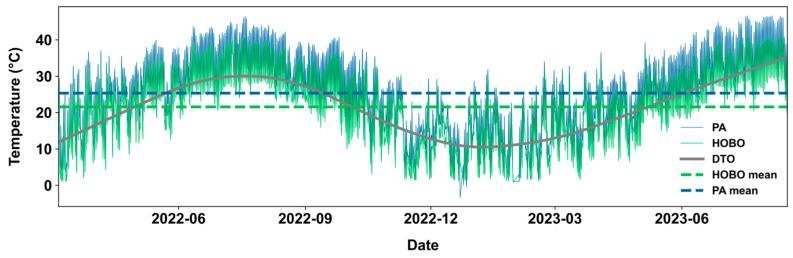
Time series of hourly mean *T_PA_* and *T_HOBO_* averaged over all stations are shown as solid curves. Dashed lines display means of the *T_PA_* and *T_HOBO_* time series. DTO represents a LOWESS curve of hourly air temperature measurements from the Denton Airport South ambient monitoring station.

The overall *MBE* is 3.77 °C during our study period, meaning that PA measurements on average overestimate ambient temperature by 3.77 °C (Figure 2). The comparison of hourly mean time series shows that *T_PA_* closely follows *T_HOBO_* but remains consistently higher, indicating a consistent overestimation. A LOWESS (locally weighted smoothing) curve has been added as a reference using air temperature measurements reported from Denton Airport South monitoring station, which has an average air temperature of 21.08 °C. Both *T_PA_* and *T_HOBO_* demonstrate similar seasonal variations with the smoothed reference temperature trend.

**Figure 3:**
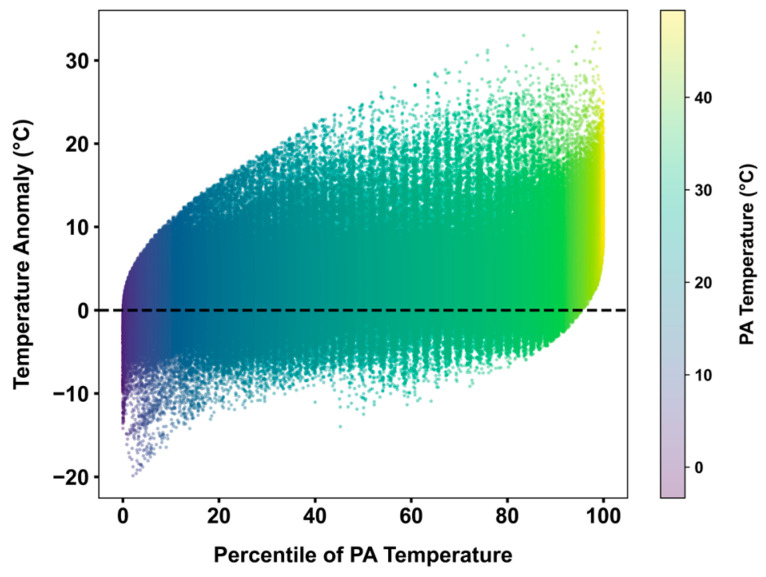
*dT* as a function of PA temperature percentile. The black dashed line represents the zero-error scenario.

Figure 3 plots *dT_i_* as a function of the percentile of *T_PA,i_* for all *n* observations. Over 79% of hourly *dT* values fall between −2 °C and 10 °C. The anomalies are particularly pronounced at the extremes, as expected, ranging from −20 °C at the lower percentiles to over +30 °C at the higher percentiles. While positive *dT* values are present in every percentile, there are no negative *dT* values at the highest percentiles. This suggests that underestimation is very rare when PA sensors report high-temperature observations (>40 °C), but underestimation and overestimation may exist when temperature is not extremely high.

*T_PA_* exhibits a moderately strong correlation with *T_HOBO_* (*r* = 0.86), but with an *MAE* and *RMSE* of 4.71 °C and 6.30 °C, respectively. The distribution of *dT* in most months is right-skewed and unimodal, except for July, which exhibits a bimodal pattern (Figure S2). January, February, August, September, and October have peaks near 0 °C, while the remaining months peak between 2 and 6 °C. This result contrasts with a previous study in Asheville, North Carolina, which reported a very strong agreement of PA (*r* = 0.99) with the reference instruments and a low overall *RMSE* of 2.8 °C [27].

**Figure 4:**
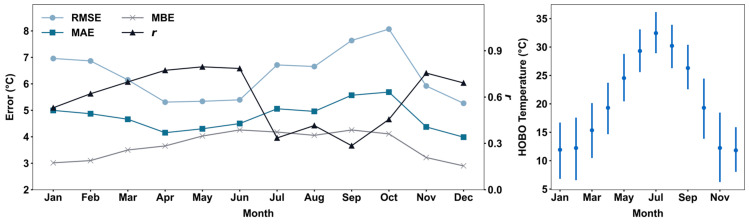
Performance metrics of hourly PA measurements for each month (**left**). Monthly mean (dot) and interquartile range (vertical line) of HOBO temperature for each month (**right**).

The performance of PA in measuring air temperature varies significantly by month, with *r* values ranging from 0.28 to 0.80 (Figure 4 left). Late summer months (July to September) exhibit a greater magnitude of overestimations when compared with winter months (December to February). May shows the highest correlation (*r* = 0.80), followed by June (0.79) and April (0.77). Conversely, September has the lowest correlation (*r* = 0.28), followed by July (*r* = 0.34). The decline in sensor performance coincides with the seasonal temperature cycle (Figure 4 right), peaking from July to September, suggesting that overheating is a primary contributor to bias in sensor readings. October has the greatest *RMSE* (8.07 °C) and *MAE* (5.69 °C), while December has the lowest *RMSE* (5.27 °C) and *MAE* (3.98 °C). The highest *MBE* (4.26 °C) is observed in June and September, while the lowest (2.90 °C) is observed in December. The *MBE* in each month stays positive, suggesting that PA tends to overestimate temperature across all seasons. The significant differences between *MAE* and *MBE* in late winter and late summer indicate the strong presence of underestimation, leading to a smaller *MBE*. Both *RMSE* and *MAE* demonstrate a significant increase starting from April, followed by a sharp decline between October and December, while *MBE* begins to increase in January and starts to decline after September. This pattern aligns with the seasonal temperature cycle, especially between spring and summer when temperature begins to increase.

**Figure 5:**
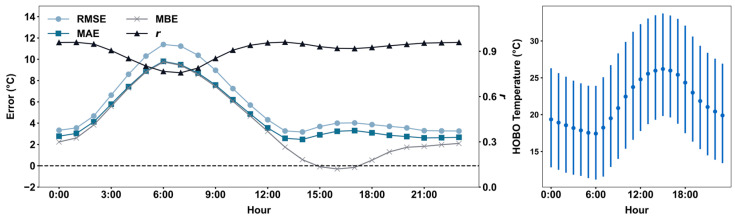
Changes in performance metrics of hourly PA measurements across different hours of the day (**left**). The black dashed line is added as a reference for overestimation and underestimation. Hourly mean (dot) and interquartile range (vertical line) of HOBO temperature for each hour (**right**).

Conversely, the diurnal performance metrics exhibit a strong antiphase relationship with diurnal temperature variations, with peak *MAE* (9.80 °C) and RMSE (11.39 °C) occurring at 6:00 AM local time, and the lowest *MAE* (2.47 °C) and *RMSE* (3.17 °C) at 2:00 PM local time (Figure 5 left). The measurement agreement does not vary as much as the monthly agreement, with *r* values ranging from 0.76 to 0.96 only. The *MBEs* are positive, except from 3:00 to 5:00 PM local time, with negative values ranging from −0.10 °C to −0.30 °C. A possible explanation for the antiphase relationship is internal heating from the WiFi module. During nighttime and early morning, when ambient temperatures are low, internal heating amplifies baseline overestimation, leading to larger temperature differences. In the afternoon, when ambient temperatures are high and PA sensors tend to underestimate the temperature, internal heating offsets this bias, resulting in higher accuracy. Regardless, PA still underestimates temperatures during peak temperature hours, leading to negative *MBEs*. Our findings highlight the nonlinear behavior of sensor performance and its diurnal and seasonal patterns, underscoring the limitation of applying a constant correction factor.

**Figure 6:**
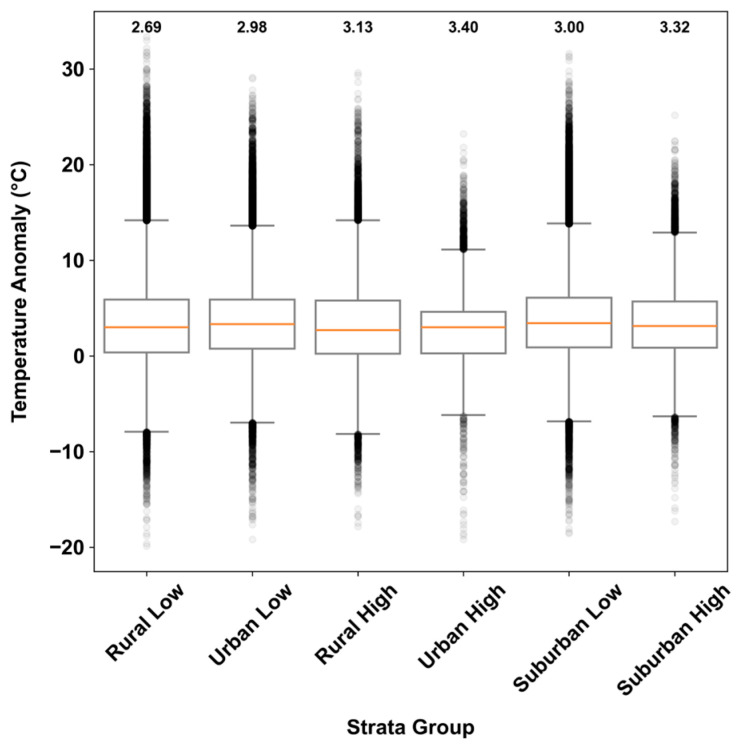
Boxplots of hourly temperature anomaly in each strata group. The median value of each group is annotated at the top.

Spatially, no systematic patterns of *dT* are identified across the six strata groups (Figure 6). All groups show similar intra-group variability except for Urban High, which has a smaller interquartile range and the highest median *dT* (3.40 °C). On the other hand, Rural Low has the lowest median *dT* (2.69 °C), followed by Urban Low (2.98 °C). The results of the Kruskal–Wallis H-test for inter- and intra-strata comparisons are statistically significant (*p* < 0.001), indicating significant differences between and within each strata group. The post hoc test shows significant differences for all strata pairs except for Rural Low and Suburban High groups. These findings suggest that sensor performance is more likely to be influenced by site-specific factors.

### 3.2. Factors Influencing Termpretuare Anomaly

**Figure 7:**
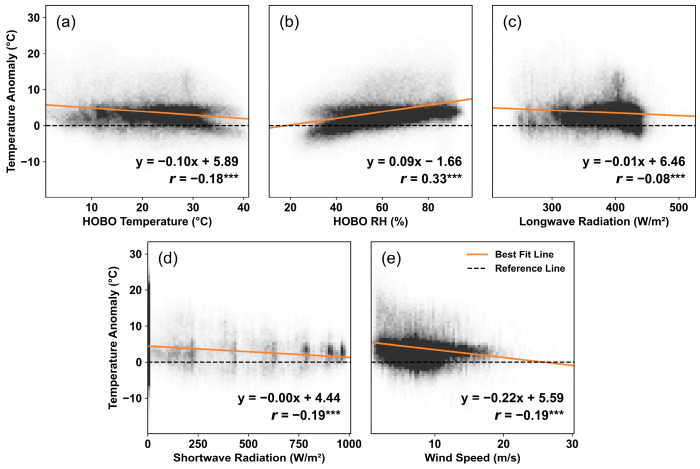
Bivariate correlation between paired hourly observations of temperature anomaly *dT*′ and (**a**) HOBO temperature, (**b**) HOBO relative humidity, (**c**) downwelling surface longwave radiation, (**d**) downwelling surface shortwave radiation, and (**e**) wind speed. The black dashed line represents the zero-error scenario, and the orange solid line is the best-fit line. *** indicates a *p*-value of <0.001.

All five meteorological variables are significantly related (*p* < 0.001) to temperature anomaly but do not exhibit strong overall correlations (|*r*| < 0.35) (Figure 7). Among them, *RH_HOBO_* has the strongest overall correlation (*r* = 0.33), while *LW* has the weakest (*r* = −0.08). At the monthly scale, all variables maintain a consistent relationship with *dT* across months. Except for *WNDS*, all meteorological variables display a downward-sloping best-fit line, indicating that PA sensors tend to overestimate temperature at lower values, with this effect diminishes as the values increase. The degree of overestimation varies by month for each variable. *T_HOBO_* has the strongest relationship in September (*r* = −0.55), while the flattest slope is observed in December (*r* = −0.25) (Figure S3). Other summer and early fall months (July, August, and October) show a similar trend, with *r* values ranging from −0.49 to −0.51.

In contrast, *RH_HOBO_* is positively correlated with *dT* across all months and follows a similar seasonal pattern (Figure S4). It has a relatively strong correlation from July to October, with *r* values ranging from 0.45 to 0.57, and the weakest correlation is found in April and June (*r* = 0.22). The similarity of the seasonal response in temperature and RH corresponds to the humid subtropical climate in Denton. In terms of solar radiation, PA sensors exhibit a high level of discrepancies when values of *SW* and *LW* are low (Figures S5 and S6). Similarly to the impact of *T_HOBO_*, they also manifest seasonal variation, with summer months showing the strongest negative correlation. From July to October, *LW* has *r* values ranging from −0.31 to −0.40, while *SW* has *r* values ranging from −0.21 to −0.33. These results are counterintuitive since we expect PA sensors to be more susceptible to radiative heating, which would lead to positive relationships with temperature anomalies. Lastly, the impact of *WNDS* also varies significantly in different months but does not exhibit a profound seasonal pattern, with *r* values ranging from −0.05 to −0.30 (Figure S7). The evident improvement in sensor performance under high wind speed conditions implies its mitigating effect on internal heating.

For the anomaly model, although the model is statistically significant (*p* < 0.001), it explains only 6.4% of the variance in *dT′* with an *R*^2^ of 0.064. All five independent variables are significantly associated with *dT′*. *WNDS′*, *LW′*, and *T_HOBO_′* show negative correlations, while *RH_HOBO_′* and *SW′* exhibit positive correlations. Their low predictive power suggests the external factors contribute to the variability of *dT′*, beyond the unexplained patterns of meteorological factors.

### 3.3. Comparison of Calibration Models for PA Temperature Sensors

The performance of the eight models generally improves incrementally as the model complexity increases (Table 2). All models have a very small *MBE*, meaning that the models do not systematically overestimate or underestimate the true values. Model 1 uses only *T_PA_* and has the worst model performance. Among the models with two additive terms (Models 2–4), Model 4 achieves the best performance with the highest *R*^2^ value (0.82) and lowest error (*RMSE* = 3.93 °C and *MAE* = 3.16 °C), outperforming models that use *RH_PA_* or *SW*. Model 7, with the most additive terms, achieves the best overall performance, followed closely by Models 5 and 6. The minimal performance improvement between Models 6 and 7, despite their added complexity with *RH_PA_* and *WNDS* terms, suggests that these predictors have limited predictive power when *LW* and *SW* additive terms are present. Model 8, which incorporates a multiplicative term to account for the collinearity between *T_PA_* and *RH_PA_*, only offers a slight improvement over Model 2, with an *RMSE* of 4.56 °C and *MAE* of 3.45 °C. Since Model 5 has very similar performance metric values as Model 6 and Model 7 but with fewer additive terms, we compared this model with Model 9, which adds its corresponding anomaly terms (*LW′* and *SW′*) to maintain the model simplicity. Model 9 shows a higher agreement (*R*^2^ = 0.89) and lower error (*RMSE* = 3.10 °C and *MAE* = 2.46 °C) compared to Model 5. All variables in Model 9 have a VIF score between 1 to 5, meaning no significant multicollinearity is found. Therefore, Model 9 is selected as the final calibration model, which generates the following equation:

(15) $$T_{HOBO}={0.2839T}_{PA}+0.117LW+0.0048SW-0.0403LW^{\prime }+0.0094SW^{\prime }-29.41$$

**Figure 8:**
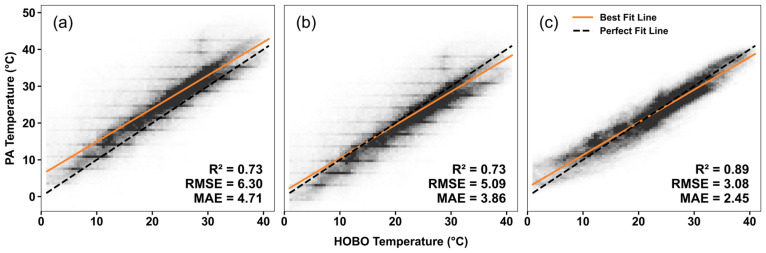
Comparison of agreement in temperature measurements with no calibration (**a**), PA-suggested calibration (**b**), and our MLR-based calibration (**c**). The black dashed line represents the perfect fit line, and the orange solid line is the best-fit line.

By comparing the calibrated *T_PA_* with the uncalibrated scenario, our model shows a reduction in *RMSE* and *MAE* by at least 51% and 47%, respectively (Figure 8). In contrast, the PA-suggested calibration only reduces *RMSE* and *MAE* by 19% and 18%. The drastic differences in their model performance highlight the strength of our MLR model in adjusting raw *T_PA_* measurements to more accurately represent true temperature conditions.

**Figure 9:**
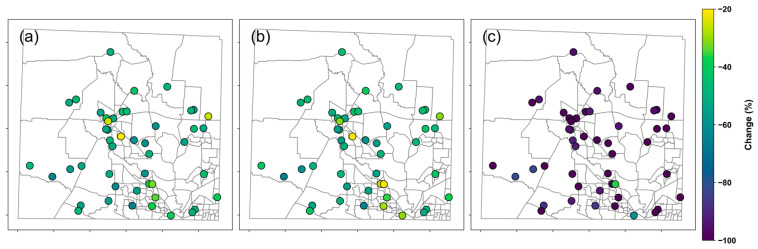
Comparison of the spatial variations of percentage change in RMSE (**a**), MAE (**b**), and MBE (**c**) after applying the MLR-based calibration. The grey lines are the census unit.

The percentage change in sensor performance following the MLR-based calibration exhibits spatial variability, with a few notable outliers (Figure 9). Overall, all sites show improvement across all performance metrics, with *MAE* demonstrating the most substantial enhancement, highlighting the effectiveness of the MLR-based calibration at the site level. The percentage change in *MBE* ranges from −36.1% to −134.5%, while *RMSE* and *MAE* show similar but much narrower ranges. Two locations in the southern part of the study area exhibit relatively small reductions in *MBE* (less than 60%) compared to other sites. Additionally, one urban site within the City of Denton shows only modest improvements, with a 14.5% reduction in *RMSE* and a 17.5% reduction in *MAE*. No obvious spatial patterns are observed in the distribution of the performance improvement. These outliers suggest the influence of site-specific factors contributing to sensor biases that are not captured by the current calibration model.

### 3.4. Evalutation in the Context of the Heat Index

**Figure 10:**
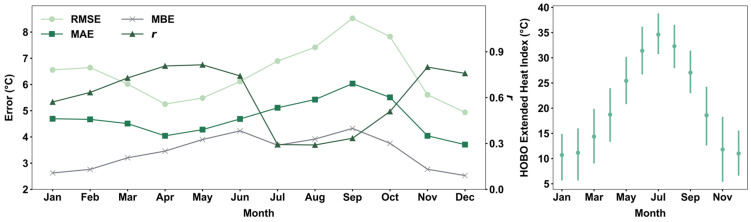
Monthly *T_PA_*-derived HI performance and *T_HOBO_*-derived HI cycle. Performance metrics of hourly *T_PA_*-derived HI measurements for each month (**left**). Hourly mean (dot) and interquartile range (vertical line) of *T_HOBO_*-derived HI for each month (**right**).

The hourly performance metrics of *T_PA_*-derived HI has a peak *MAE* and *RMSE* value recorded at 6:00 AM local time, and the lowest at 1:00 PM local time (Figure S8), demonstrating similar diurnal patterns with the performance of *T_PA_*. However, there are strong differences between their monthly performances, particularly in sensor agreement in August, in which *T_PA_*-derived HI (*r* = 0.29) demonstrates a much weaker agreement than *T_PA_* (*r* = 0.42) (Figure 10). Similar to the monthly performance pattern of T_PA_, there is a steady increase in the magnitude of overestimation from May to September, with the highest *RMSE* (8.52 °C), *MAE* (6.03 °C), and *MBE* (4.32 °C) in September. However, the sharp improvement in sensor performance begins in September instead of October, with the lowest *RMSE* (4.94 °C) and *MAE* (3.71 °C), and *MBE* (3.71 °C) observed in December. HI shows a greater magnitude of overestimation with the largest *RMSE* (0.89 °C) and *MAE* difference (0.46 °C) with *T_PA_* performance metrics in September. The results exemplify the possibility of having a compounding effect where the mixture of *RH_PA_* and *T_PA_* measurements amplifies the magnitude of overestimation.

Table 3 summarizes the proportion of hourly temperature measurements in each heat class after converting hourly *T_HOBO_* and *T_PA_* to hourly HI. Similar to ambient temperature, PA tends to overestimate apparent temperature, and it is most significant in the Extreme Caution and Danger class, in which they are 6.3% and 5.7% higher than *T_HOBO_*-derived HI respectively. However, the discrepancy is relatively small in the Caution and Extreme Danger class, illustrating its vulnerability in certain heat classes. The differences highlight the shortcomings of a potential application of PA meteorological data as it may raise a false alarm for heat events due to inherent temperature overestimation.

## 4. Limitations

Generality is a key criteria for a calibration model, as it ensures the model can operate across different geographical locations and seasons [47]. To maintain generality and simplicity, we considered only a linear relationship. While most regression model results in our study area show a high degree of linearity, this may not apply to other regions. For example, some studies discovered a nonlinear effect of *RH_PA_* [48] on PA particulate matter measurements and applied a nonlinear empirical correction equation [49]. Therefore, it is necessary to test various regression functions to account for nonlinearity. Additionally, this study used temperature data from only one county. As a result, the model’s transferability may not be applicable to other climate zones with significantly different local climates.

While the sensor distribution covered the study area well and a stratified sampling approach was used to reduce bias, some strata were oversampled and others were under-sampled. For instance, Suburban High only had one site (3.6% of the dataset), but Suburban Low had 16 sites (36.9% of the dataset). This imbalance was also constrained by the recruitment timeline. Furthermore, this stratified sampling approach only considered two urban factors to characterize the built environment. However, the urban thermal environments are heterogeneous in both horizontal and vertical dimensions, influenced by complex anthropogenic and natural factors [50]. Consequently, other classification systems, such as the Local Climate Zone [51], may be more effective in representing different local climates, capturing systematic patterns, and optimizing model development. Moreover, the spatial analysis of PA temperature performance was limited to the strata grouping approach, as site-specific analysis was not feasible due to data incompleteness and significant imbalances in sample sizes across sites. A more robust and complete dataset is needed to enable reliable spatial assessments at the local level.

In terms of independent variable selection, this study focused on PA sensitivity to meteorological factors and did not consider other mechanical factors, such as sensor age. Since the life expectancy of a PA sensor is about two years, aging sensors can degrade and cause significant inaccuracies. To address this, some studies have incorporated the sensor’s total operating time and the consecutive operation time in their calibration models to account for sensor aging and operational stability [37]. Including these factors could improve the calibration model’s performance, but since not all sensors have this information available, we opted not to include them to reduce complexity.

## 5. Conclusions

This study provides a firsthand and comprehensive assessment of PA temperature measurements and proposes a linear calibration model. Uncalibrated PA temperature measurements had a moderate agreement with reference data (r = 0.86) but overestimated temperature by 3.77 °C on average. The results reveal seasonal and diurnal variations in PA sensor performance, influenced by local conditions. The selected calibration model outperformed the PA-suggested calibration method, achieving *MAE* = 2.45 °C and *RMSE* = 3.08 °C, providing a simple yet effective adjustment for PA data. While all eight models performed well, the improvements in accuracy were relatively small compared to the additional effort required to collect more variables—particularly radiation variables. For general use, it is important to balance the benefits of slightly improved accuracy with the practicality of data collection. The Heat Index application highlights PA’s potential in heat monitoring, though data accuracy is crucial to avoid overestimating heat events. As rising temperatures threaten our public health and urban livability, reliable in situ temperature data from LCSs, like PA, become valuable. This work underscores the importance of leveraging PA’s network for heat monitoring and offers guidance for integrating such data into research and urban decision-making processes.

## Figures and Tables

**Table 1:** Descriptions of meteorological variables used in calibration model development.

Variable (Acronym)	Source	Unit	Spatial Resolution	Temporal Resolution
Wind speed (*WNDS*)	Texas Commission on Environmental Quality	m/s	/	Hourly
Longwave downwelling surface irradiance (*LW*)	GOES-East Surface Solar Irradiance	W/m^2^	0.05°	
Shortwave downwelling surface irradiance (*SW*)	GOES-East Surface Solar Irradiance	W/m^2^	0.05°	
Relative Humidity (*RH_PA_*)	PurpleAir Sensor	%	/	
Air Temperature (*T_PA_*)	PurpleAir Sensor	°C	/	

**Table 2:** Comparison of calibration model performance.

Model	Number of Training Sample	*R* ^2^	*RMSE* (°C)	*MAE* (°C)	*MBE* (°C)
1	209,048	0.73	4.73	3.54	0.01
2	209,048	0.75	4.59	3.49	0.01
3	209,048	0.77	4.41	3.38	0.01
4	208,988	0.82	3.93	3.16	−0.02
5	208,988	0.86	3.38	2.73	0
6	208,988	0.86	3.38	2.73	0
7	207,999	0.87	3.37	2.72	0.01
8	209,048	0.75	4.56	3.45	0.01
9	208,988	0.89	3.10	2.46	−0.01

**Table 3:** Comparison of Heat Index classification derived from PA and HOBO data.

Class	Range of HI (°F)	HOBO HI (% of Time)	PA HI (% of Time)
Caution	80–90	17.6	18.8
Extreme Caution	90–103	14.0	20.3
Danger	103–124	3.9	9.6
Extreme Danger	≥125	0	0.3

## Data Availability

The field calibration code and data for PurpleAir temperature measurements are available at https://github.com/lu-liang-geo/PurpleAir-Temp-Calibration/tree/main (accessed on 5 May 2025).

## Supplementary Materials

The following supporting information can be downloaded at: https://www.mdpi.com/article/10.3390/s25103044/s1, Table S1: Classification rules of each sampling strata; Figure S1: Collocation of a PurpleAir sensor and a HOBO data logger. Figure S2: Monthly histograms of temperature anomaly; Figure S3: Monthly scatterplots of hourly temperature anomaly and hourly HOBO temperature; Figure S4: Monthly scatterplots of hourly temperature anomaly and hourly HOBO relative humidity measurements; Figure S5: Monthly scatterplots of hourly temperature anomaly and hourly downwelling longwave irradiance observations; Figure S6: Hourly temperature anomaly and hourly downwelling shortwave irradiance observations; Figure S7: Monthly scatterplots of hourly temperature anomaly and hourly wind speed measurements; Figure S8: Monthly T_PA_-derived Heat Index performance and T_HOBO_-derived Heat Index temperature cycle.

## Abbreviations

The following abbreviations are used in this manuscript:

LCS	Low-cost sensors
PA	PurpleAir
*MAE*	Mean absolute error
*RMSE*	Root mean square error
AIC	Akaike information criterion
*RH*	Relative humidity
*WNDS*	Wind speed
*LW*	Longwave downwelling surface irradiance
*SW*	Shortwave downwelling surface irradiance
UHI	Urban heat island
HI	Heat Index
MLR	Multiple linear regression
VIF	Variance inflation factor
