# Supplementary material for: Evaluating PurpleAir Sensors: Do They Accurately Reflect Ambient Air Temperature?
Source: Sensors (Basel). 2025 May 12;25(10):3044. doi: 10.3390/s25103044 (PMC12114826; doi:10.3390/s25103044)
Supplement: Supplementary file 1 [file sensors-25-03044-s001.zip › sensors-3562890-supplementary.pdf]

Supplementary Material

Evaluating PurpleAir Sensors: Do They Accurately Reflect Ambient Air Temperature?

Justin Tse [1] and Lu Liang [1,*]

[1] Department of Landscape Architecture and Environmental Planning, University of California, Berkeley, CA 94720, USA
* Correspondence: lianglu@berkeley.edu

Number of Tables: 1
Number of Figures: 8

Table S1. Classification rules of each sampling strata.

Sampling strata	Impervious surface (%)	Distance of grid center to major roads (meter)
Urban High	>50	<150
Urban Low	>50	>150
Suburban High	20-50	<150
Suburban Low	20-50	>150
Rural High	<20	<150
Rural Low	<20	>150

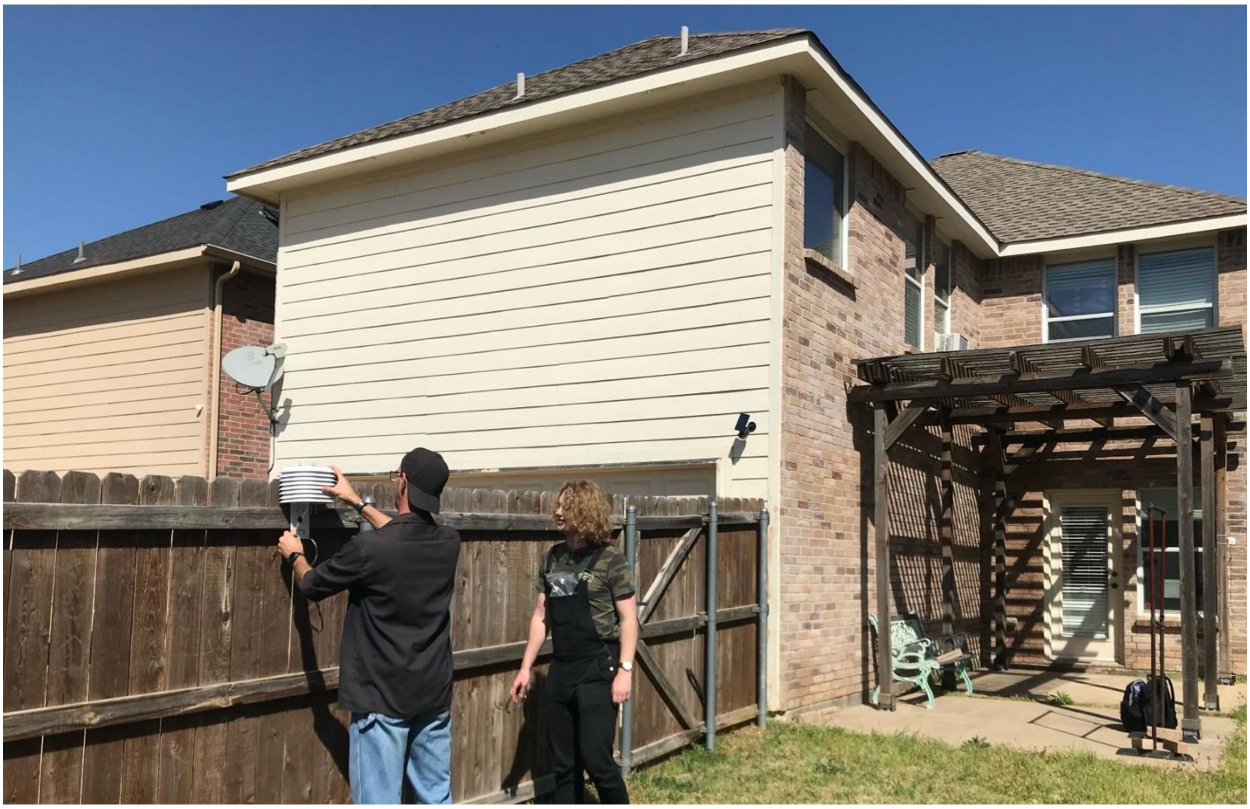

Figure S1. A typical setup showing the colocation of a PurpleAir sensor and a HOBO data logger at a residential property.

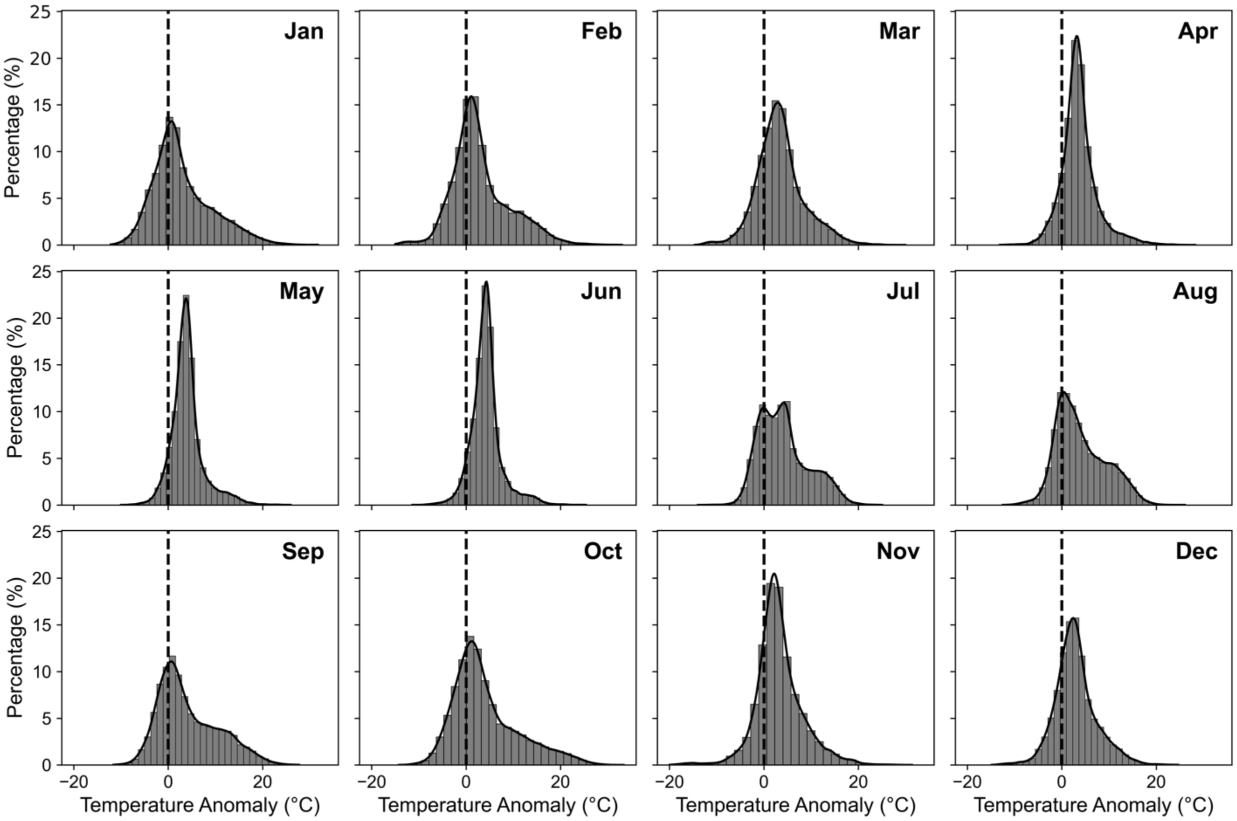

Figure S2. Monthly histograms of temperature anomaly. The black dashed line represents the zero-error scenario.

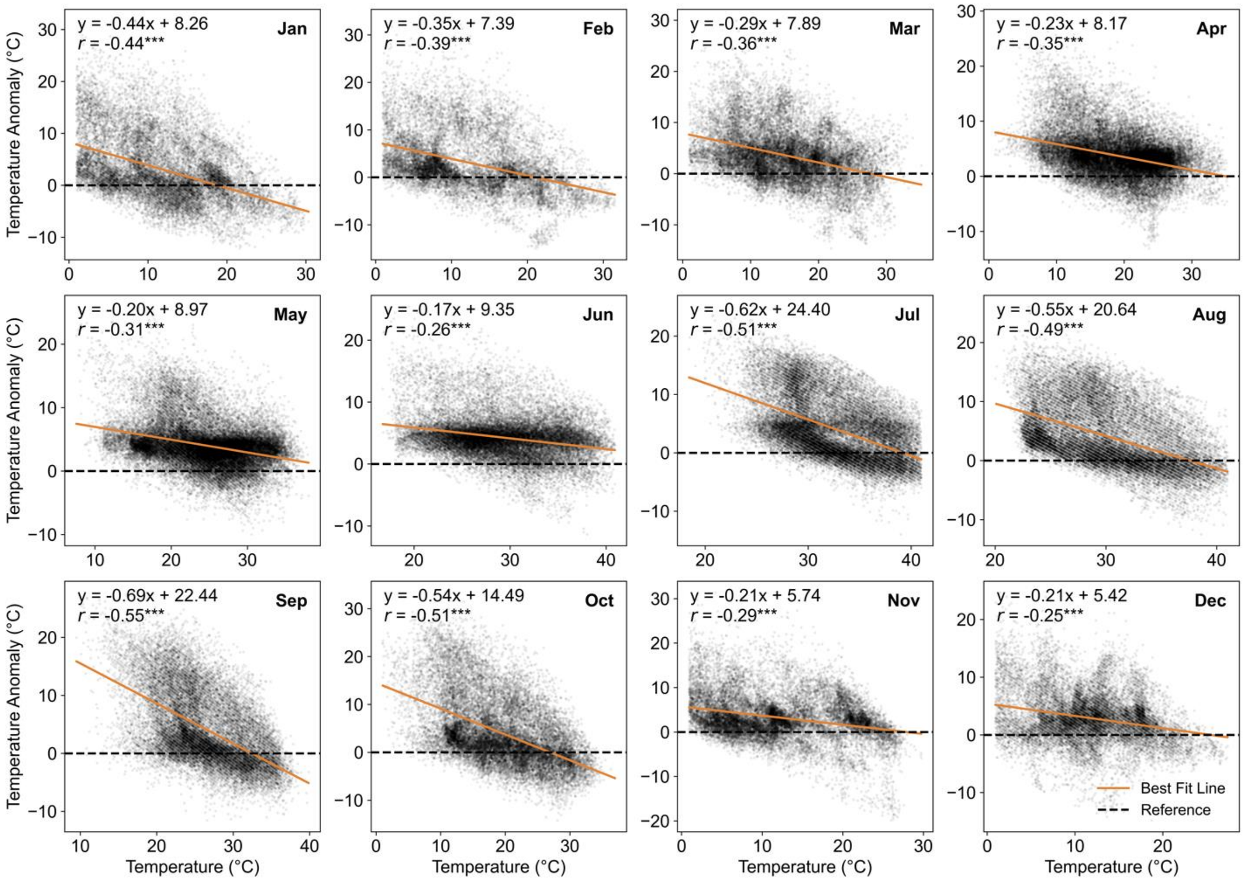

Figure S3. Monthly scatterplots of hourly temperature anomaly and hourly HOBO temperature measurements. The black dashed line represents the zero-error scenario, and the orange solid line is the best-fit line. Level of significance is indicated by * ($p < 0.05$), ** ($p < 0.01$), *** ($p < 0.001$).

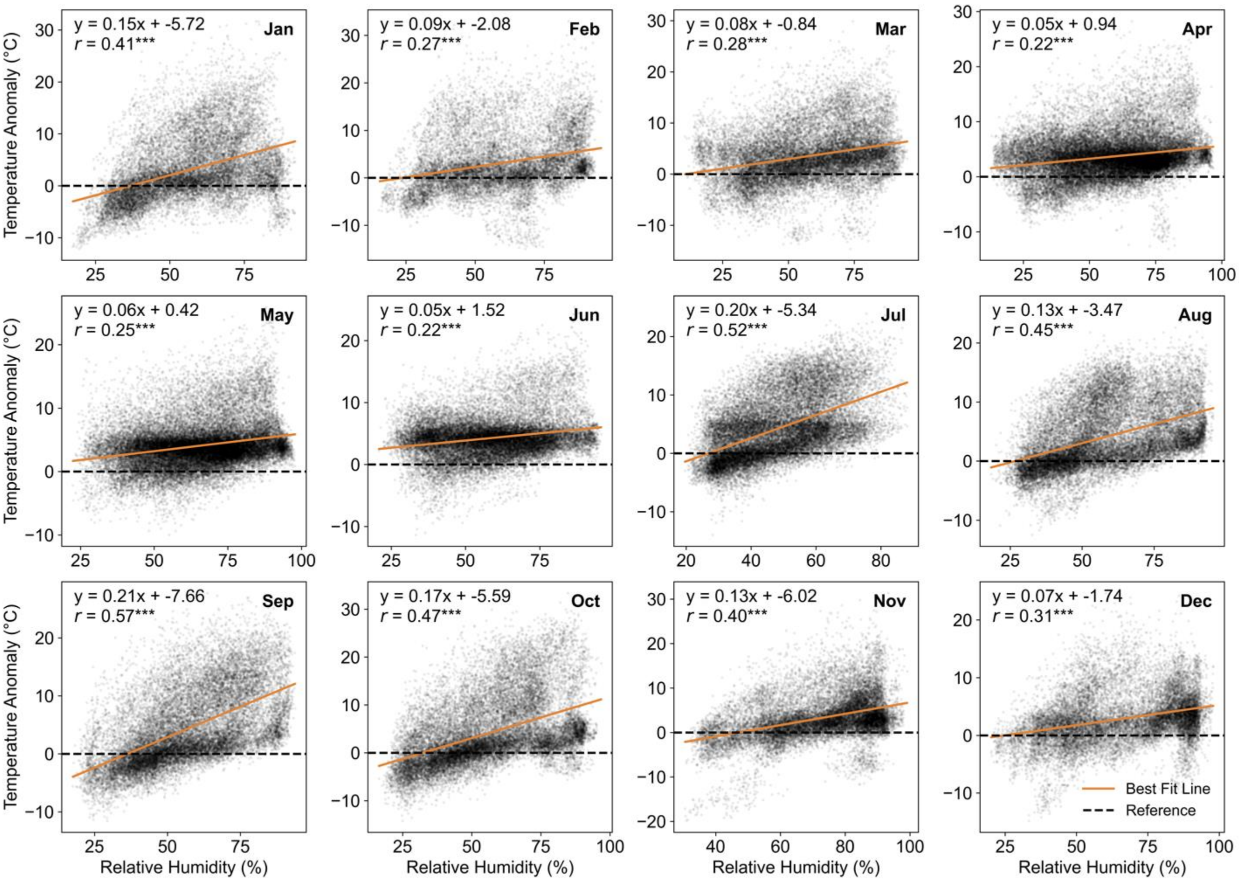

Figure S4. Monthly scatterplots of hourly temperature anomaly and hourly HOBO relative humidity measurements. The black dashed line represents the zero-error scenario, and the orange solid line is the best-fit line. Level of significance is indicated by * ($p < 0.05$), ** ($p < 0.01$), *** ($p < 0.001$).

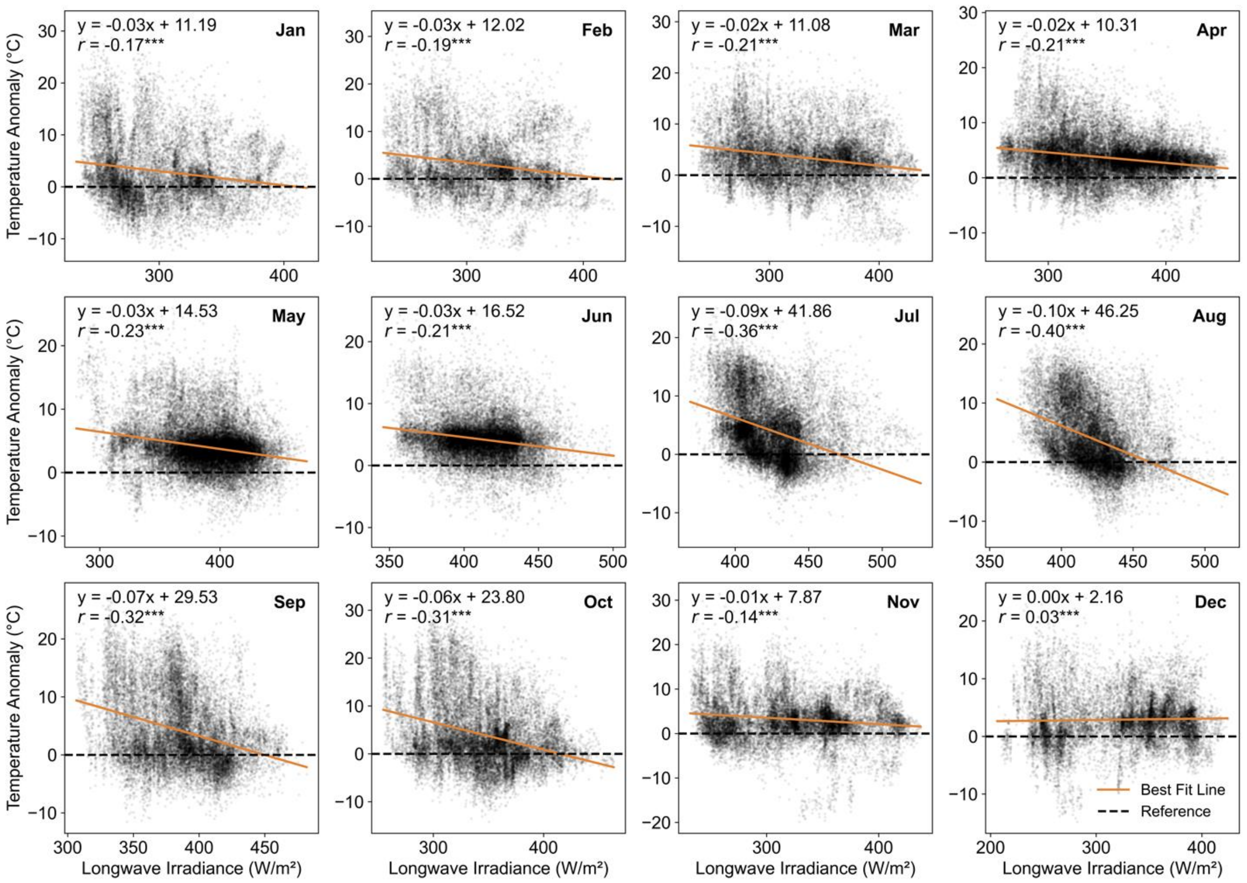

Figure S5. Monthly scatterplots of hourly temperature anomaly and hourly downwelling longwave irradiance observations. The black dashed line represents the zero-error scenario, and the orange solid line is the best-fit line. Level of significance is indicated by * ($p < 0.05$), ** ($p < 0.01$), *** ($p < 0.001$).

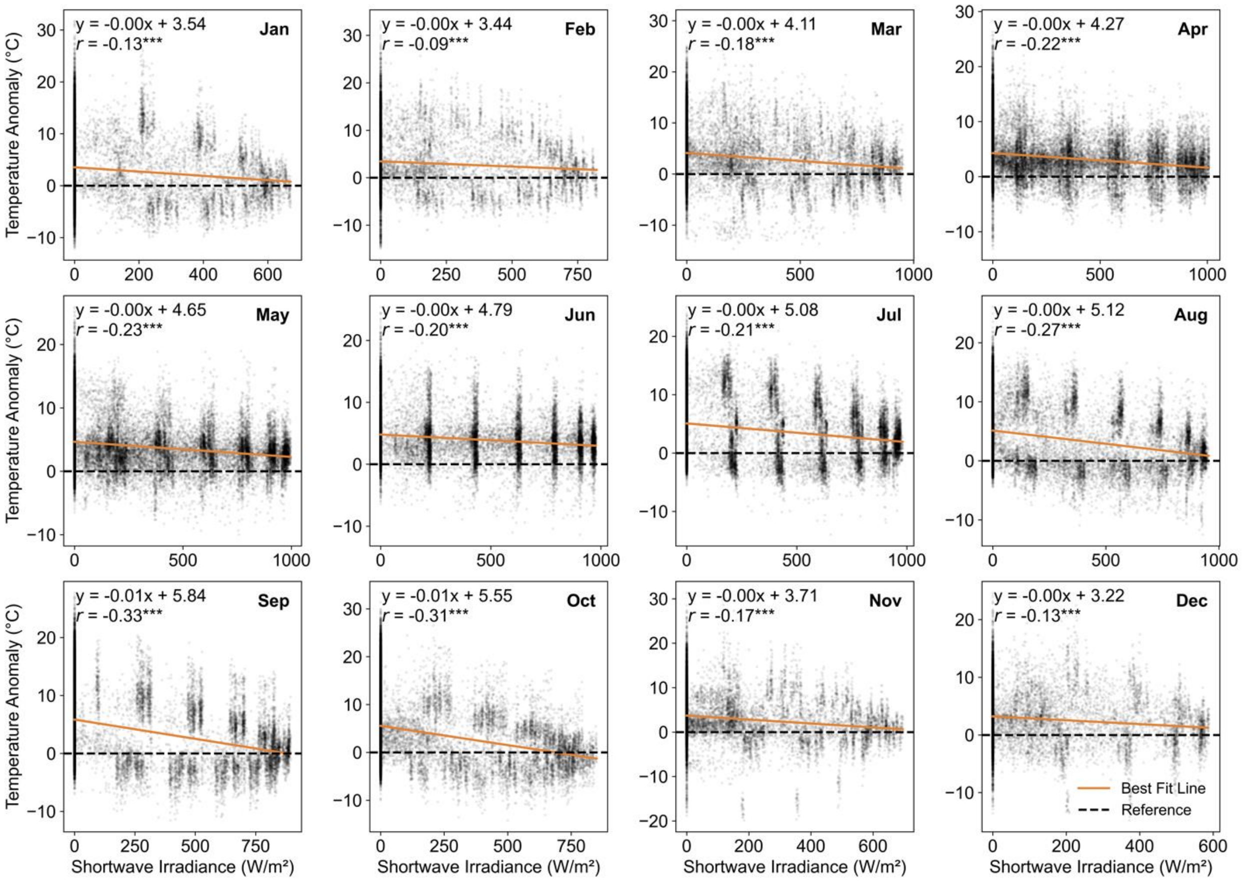

Figure S6. Monthly scatterplots of hourly temperature anomaly and hourly downwelling shortwave irradiance observations. The black dashed line represents the zero-error scenario, and the orange solid line is the best-fit line. Level of significance is indicated by * ($p < 0.05$), ** ($p < 0.01$), *** ($p < 0.001$).

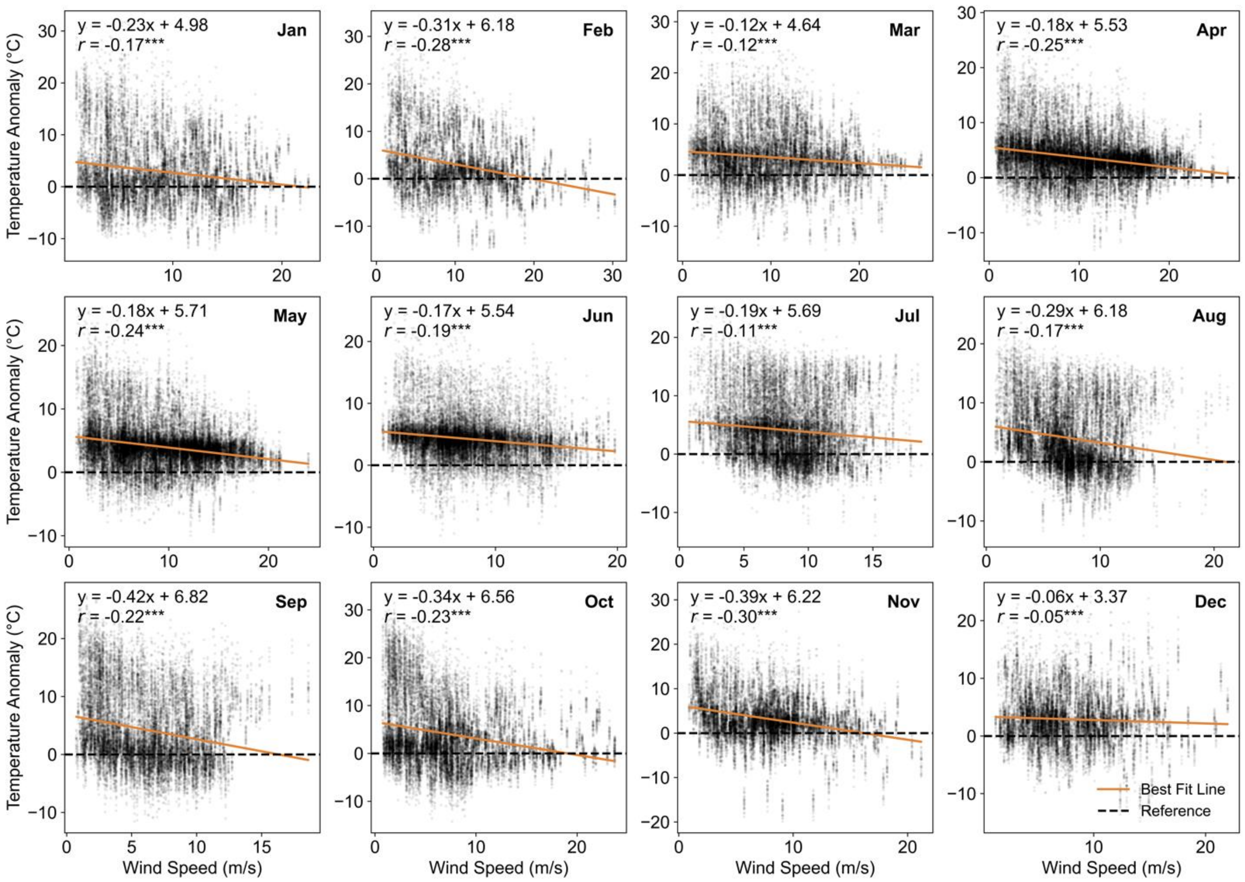

Figure S7. Monthly scatterplots of hourly temperature anomaly and hourly wind speed measurements. The black dashed line represents the zero-error scenario, and the orange solid line is the best-fit line. Level of significance is indicated by * ($p < 0.05$), ** ($p < 0.01$), *** ($p < 0.001$).

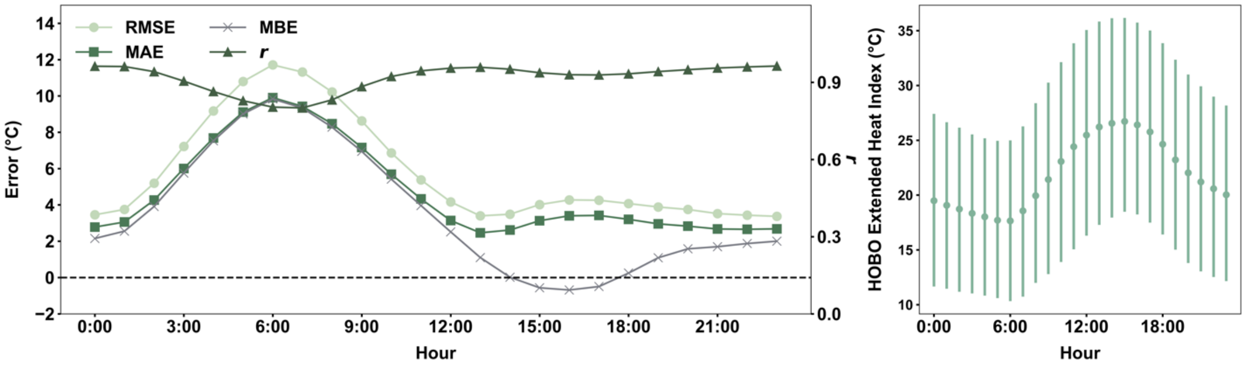

Figure S8. Hourly T_{PA}-derived Heat Index performance and T_{HOBO}-derived Heat Index temperature cycle. Performance metrics of hourly T_{PA}-derived HI measurements for each hour (left). The black dashed line is added as a reference for overestimation and underestimation. Hourly mean (dot) and interquartile range (vertical line) of T_{HOBO}-derived HI temperature for each hour (right).
